# Health related quality of life and parental perceptions of child vulnerability among parents of a child with juvenile idiopathic arthritis: results from a web-based survey

**DOI:** 10.1186/1546-0096-12-34

**Published:** 2014-08-07

**Authors:** Lotte Haverman, Hedy A van Oers, Heleen Maurice-Stam, Taco W Kuijpers, Martha A Grootenhuis, Marion AJ van Rossum

**Affiliations:** 1 Psychosocial Department, Academic Medical Center, Emma Children’s A3-241 Hospital, Postbox 22660, Amsterdam 1100 DD, the Netherlands; 2 Department of Pediatric Hematology, Immunology and Infectious Diseases, Academic Medical Center, Emma Children’s Hospital, Amsterdam, the Netherlands; 3 Department of Pediatric Hematology, Immunology and Infectious Diseases, and Reade (Location Jan van Breemen), Department of Pediatric Rheumatology, Amsterdam, the Netherlands

**Keywords:** Parents, Child vulnerability, Psychosocial factors, Health related quality of life, Juvenile idiopathic arthritis, Caregivers, Pediatrics, Disease activity

## Abstract

**Background:**

A chronic illness, such as Juvenile Idiopathic Arthritis (JIA), has an impact on the whole family, especially on parents caring for the ill child. Therefore the aim of this study is to evaluate parental Health Related Quality of Life (HRQOL) and parental perceptions of child vulnerability (PPCV) and associated variables in parents of a child with JIA.

**Methods:**

Parents of all JIA patients (0–18 years) in Amsterdam, the Netherlands, were eligible. HRQOL was measured using the TNO-AZL Questionnaire (TAAQOL) and PPCV using the Child Vulnerability Scale (CVS). The HRQOL of parents of a child with JIA was compared to a norm population, and differences between parents of a child with JIA and active arthritis versus parents of a child with JIA without active arthritis were analyzed (ANOVA). For PPCV, parents of a child with JIA were compared to a norm population, including healthy and chronically ill children (Chi^2^, Mann-Whitney U test). Variables associated with PPCV were identified by logistic regression analyses.

**Results:**

155 parents (87.5% mothers) completed online questionnaires. JIA parents showed worse HRQOL than parents of healthy children on one out of twelve domains: fine motor HRQOL (p < .001). Parents of children with active arthritis showed worse HRQOL regarding daily activities (p < .05), cognitive functioning (p < .01) and depressive emotions (p < .05) compared to parents of children without active arthritis. Parents of children with JIA perceived their child as more vulnerable than parents of a healthy child (p < .001) and parents of a chronically ill child (p < .001). Parents of children with active arthritis reported higher levels of PPCV (p < .05) than parents of children without active arthritis. A higher degree of functional disability (p < .01) and shorter disease duration (p < .05) were associated with higher levels of PPCV.

**Conclusion:**

The HRQOL of JIA parents was comparable to the HRQOL of parents of a healthy child. JIA parents of a child with active arthritis showed worse HRQOL than parents of a child without active arthritis. Parents perceived their child with JIA as vulnerable.

## Background

Juvenile Idiopathic Arthritis (JIA) is arthritis of unknown etiology that starts before the age of sixteen. It is one of the most common rheumatic diseases in childhood and a major cause of childhood disability. Worldwide, 0.07-4.01 per 1000 children are affected [1]. Children suffering from JIA experience functional impairment due to manifestations of the disease in one or more joints, morning stiffness and fatigue [2]. In addition, children with JIA are more functionally disabled and experience more pain than healthy children, especially children with the diagnosis of JIA and active arthritis [1,3]. There is no definite cure for JIA: treatment is aimed at controlling pain and achieving inactive disease by means of medication [1].

A chronic illness has an impact on the whole family, especially on parents caring for the ill child [4]. Parents of a chronically ill child frequently report mood problems, anxiety, physical problems, cognitive problems and a feeling of lack of control over daily events because of their child’s illness [5]. They are also more likely to report higher levels of distress and lower levels of Health Related Quality of Life (HRQOL) than parents of healthy children [6,7]. Parenting a child with a chronic illness affects the roles of parents, such as being a partner, employee or family member [8-11] ultimately resulting in increased burden for parents [12-14]. Moreover, high levels of distress in parents are associated with the child’s maladjustment to the illness [15-18].

Like other chronic illnesses, caring for a child with JIA may have many adverse social and emotional consequences for the entire family [19]. It is plausible that parents of a child with JIA do experience psychosocial problems or a worse HRQOL [20]. The psychosocial impact of JIA on parents has been documented only to a limited extend. Some results suggest that mothers of a child with JIA experience increased feelings of depression [21,22], show increased number of mental health problems [23] and increased psychological distress [24]. However, other studies do not find any differences between parents of a child with JIA and comparison groups on parental distress [25], nor on HRQOL, anxiety and depression [26]. In addition, it remains unclear what the influence is of disease activity, functional ability or pain of children with JIA on parental functioning or HRQOL [19,20,23,24,26]. Because little is known about these parents, it is important to gain more insight in the parental psychosocial functioning. The parent’s well-being affects the well-being of the child [27].

Furthermore, a parent’s belief that a child is vulnerable can potentially influence their child’s development [28]. Children with a chronic illness tend to be more socially anxious when their parents perceive them as more vulnerable [29]. In addition, high perceived vulnerability can lead to overprotective behavior in parents, to more parental stress, and to psychological problems in children, such as psychosomatic complaints and school underachievement [30,31]. Parents who believe that their child is vulnerable to illness were more likely to keep their children home from school [32]. In addition, increased disease severity and lower parental educational level were associated with higher Parental perceptions of Child vulnerability (PPCV) [29].

The aims of this study are (1) to evaluate the HRQOL measured with the TNO-AZL Questionnaire for Adult’s Health Related Quality of Life (TAAQOL) of parents of a child with JIA compared to a Dutch TAAQOL norm group and (2) to compare parents of children with the diagnosis JIA with and without active arthritis on HRQOL, (3) to evaluate the PPCV measured with the Child Vulnerability Scale (CVS) among parents of a child with JIA compared to the Dutch CVS norm group existing of parents with a healthy child and parents with a child with another chronic health condition, (4) to compare parents of children with the diagnosis JIA with and without active arthritis on PPCV, and (5) to identify which parental and child variables are associated with PPCV.

## Methods

### Participants

In this study, we collected data from parents of a child (one parent per child) who consulted a pediatric rheumatologist between February 2009 and March 2010 at one of the four referral centers in Amsterdam: the Emma Children’s Hospital/AMC, VU Medical Centre, Reade (location Jan van Breemenstraat) and Sint Lucas Andreas Hospital. Parents of a child (aged 0–18 years) with JIA were eligible. Before a planned outpatient consultation, a letter was sent to the parents and patients, which set out the purpose and the procedure of the study. All parents gave informed consent according regulations and the study was approved by the Medical Ethics Committees of the participating centers. A username and password were sent by e-mail to the participating patients enabling them to log in to the study website providing the online questionnaires. Eligible parents of JIA patients attending the outpatient clinic during the study period who did not complete the questionnaires, were defined as non-participants.

### Measures

The following concepts were measured; socio-demographics, medical data, functional ability, Health Related Quality of Life and parental perceptions of child vulnerability.

### Socio-demographics and medical data

#### Socio-demographics

Socio-demographics on participating children and parents were collected using online baseline questionnaires completed by the mother or father. The following information concerning the child was obtained: age, age at onset of disease and gender. Children completed a questionnaire about their subjective burden of medication use. Data were obtained from the parents on their age, gender, country of birth, education (low: no education, primary school or primary vocational education; middle: secondary school or secondary vocational education; high: higher vocational education or university), number of children and marital status. Socio-demographic information on the non-participating parents was missing, except the country of birth, because this was registered in the medical files.

#### Medical data

The medical data of the participating children were assessed by the pediatrician during the consultation. The medical data of the non-participating children were collected retrospectively, based on the information in the medical files. All patients were classified (JIA type) according to the International League of Associations for Rheumatology (ILAR) criteria [33]. During the consultation, the physician assessed the disease activity on a 100 mm Visual Analogue Scale (VAS) (0 = no disease activity; 100 = very severe activity) and the number of active joints. These were classified as follows: no active joints (no active arthritis), monoarthritis (1 joint), oligoarthritis (2 to 4 joints), polyarthritis (5 to 10 joints), and severe polyarthritis (11 or more active joints). The last four categories were regarded as ‘children with active arthritis’. The patient’s medication at the time of the consultation was recorded. Current or previous presence of uveitis was noted. The time between disease onset and diagnosis was calculated as well as disease duration (time from disease onset to the date of the consultation).

### Functional ability and discomfort

#### CHAQ

The Dutch version of the Childhood Health Assessment Questionnaire (CHAQ) [34] was used to measure functional ability [35]. The disability index is a summarized score ranging from 0 to 3, with higher scores indicating higher disability. The CHAQ can be used as a self-report as well as a parent proxy report. In this study, both versions were used: the proxy report (ages 0 to 7 years) and the self-report (ages 8 to 18 years).

#### Discomfort

Discomfort was assessed by a 100 mm VAS for the evaluation of pain (0 = no pain; 100 = very severe pain) and a 100 mm VAS for the evaluation of overall well-being (0 = very well; 100 = very poor), completed by the parent (ages 0 to 7 years) or patient (ages 8 to 18 years).

### Health Related Quality of Life with the TAAQOL

Parental HRQOL was assessed by the TNO-AZL Questionnaire for Adult’s Health Related Quality of Life (TAAQOL) [36]. The questionnaire was completed by parents. This questionnaire measures health status problems weighted by the impact of problems on well-being on 12 multi-item scales, with higher scores indicating better HRQOL. Each item consists of 2 parts: the first part assesses the prevalence of a health problem or limitation in the past month, and the second part evaluates the emotional response to the health problem or limitation. Answers were scored on 4-point scales. A single score is attributed to each combination of an item assessing the prevalence of a problem or limitation and the corresponding emotional response. The psychometric properties, validity, and reliability of the TAAQOL were satisfactory [36]. The Cronbach’s α values in the present study sample were good, ranging from .71 to .92. The norm group consists of parents of healthy children from two Dutch elementary schools and one high school, located in Amsterdam [7].

### Parental perceptions of child vulnerability with the CVS

Perceptions of child vulnerability by mother or father were assessed using the Child Vulnerability Scale (CVS) [28]. The questionnaire was completed by parents. The CVS was translated into Dutch [37]. The CVS consists of eight items with a 4-point response scale ranging from ‘definitely false’ to ‘definitely true’ scored from 0 to 3, with a total score of 0 to 24. A score equal to or greater than 10 is the cut-off for high vulnerability (defined as scores in the clinical range) [28]. The internal consistency of the Dutch scale was good, with a Cronbach’s α value of .70 [37]. The norm group consists of 520 parents of children from 9 Dutch schools. The presence of a chronic health condition was proxy reported. This group (n = 69) included: asthma (36.2%), congenital defect (13.0%), skin disease (5.8%) and migraine (5.8%) [37]. The Cronbach’s α in the present study sample was .83.

### Statistical analyses

The Statistical Package for the Social Sciences (SPSS) version 19.0 was used to manage and analyze the data. First, differences between participants and non-participants were analyzed, using independent samples t-test, Mann–Whitney U test or Chi^2^ test.

Second, the TAAQOL scores (HRQOL) were computed according to the manual. HRQOL differences between (1) the parents of a child with JIA and the Dutch norm group, as well as between (2) parents of a child with the diagnosis of JIA without active arthritis (no arthritis) and parents of a child with the diagnosis of JIA with active arthritis (monoarthritis, oligoarthritis, polyarthritis or severe polyarthritis) were analyzed with analysis of variance (ANOVA), corrected for the differences between the groups on socio-demographics, using independent samples t-test, Mann–Whitney U test or Chi^2^ test to analyze the differences.

Third, the CVS (parental perceptions of child vulnerability, PPCV) total score was computed according to the manual and the answers on the individual CVS items were dichotomized (0 = definitely true and mostly true, 1 = definitely false and mostly false). Differences on the PPCV between the parents of a child with JIA and both norm groups (healthy and chronically ill) were analyzed with Mann Whitney U tests for the total score and Chi^2^ tests for the scores in the clinical range (scores ≥10) and on the dichotomized item level. The tests mentioned above are also used to study differences in parents of children with JIA with and without active arthritis. It was not necessary to correct the CVS analyses for parental educational level because this characteristic appeared nor to be correlated with the CVS scores in the norm group [37] neither in the parents of children with JIA (correlations .003-.097). Ethnicity was correlated with CVS in the norm group [37] but the groups to be analyzed in the present study did not differ on this characteristic.

Finally, logistic regression analyses were performed to examine which parental and child variables were associated with PPCV, expressed by a score equal or above the cut-off score of 10. First, univariate logistic regression analyses were performed on possible predictive variables. All variables that showed significant (p < .05) associations with PPCV in the univariate logistic regression analyses were included in the final logistic regression model of PPCV simultaneously, i.e. parental age, number of active joints, disease duration, functional ability (CHAQ total score) and patient and parent reported pain (VAS scores). Parental gender, parental country of birth, parental education, child’s age, child’s gender, diagnosis, medication use, subjective burden of medication use were therefore excluded. Owing to multicollinearity (high correlation between predictors, correlation > .80), general well-being (VAS score) and physician disease activity score (VAS score) were also excluded.

## Results

### Socio-demographic and medical information

237 parents of children with JIA were approached to participate. Parents and children (above the age of 12) had to give their permission to participate. 168 (61.5%) of these parents completed the online HRQOL questionnaire and CVS questionnaire. The main reason for parents and children, especially adolescents, not to participate was because they did not feel like they wanted to participate, or because of a lack of time.

A total of 155 parents completed the socio-demographic questionnaire; 87.5% of the parents were mothers and the mean age was 41.99 (SD = 5.40). In 83.7%, the parental country of birth was the Netherlands. The distribution of parental educational level was as follows: 13.1% low, 51.2% middle, 35.7% high. 67.3% of the participating children were female, and the mean age of all children was 11.48 years (SD = 4.6) (Table 1).

**Table 1:** Socio-demographics and disease characteristics of participants and non-participants

	***Participants***			***Non-participants***			
**PARENTS**	**N**	**M**	**SD**	**N**	**M**	**SD**	
Age (years)	155	41.99	5.40				
	**N**	**%**		**N**	**%**		**p**
Country of birth parents (Netherlands)	139	83.7		65	61.9		<.001^b^
Gender (female)	147	87.5					
Married/living together	158	94.0					
Education							
Low	22	13.1					
Middle	86	51.2					
High	60	35.7					
Number of children							
1	22	13.1					
2-3	135	80.3					
> 3	11	6.6					
**CHILDREN**	**N**	**M**	**SD**	**N**	**M**	**SD**	**p**
Age (years)	168	11.48	4.56	105	13.28	3.87	<.001^a^
Age of disease onset	165	7.40	4.42	104	8.61	4.46	.03^a^
Age of diagnosis	168	8.58	4.69	103	9.72	4.52	.05^a^
	**N**	**%**		**N**	**%**		**p**
Gender (female)	113	67.3		65	61.9		.34^b^
JIA subtype	**N**	**%**		**N**	**%**		**p**
Oligo-articular JIA, persistent	40	13.8		19	18.1		.26^b^
Oligo-articular JIA, extended	21	12.5		12	11.4		.79^b^
Poly-articular JIA, RF negative	66	39.4		46	43.8		.46^b^
Poly-articular JIA, RF positive	5	3.0		4	3.8		.71^b^
Systemic JIA	7	4.2		3	2.9		*
Enthesitis related Arthritis	18	10.7		11	10.5		.95^b^
Undifferentiated JIA	9	5.3		8	7.7		.50^b^
Chronic arthritis with other autoimmune inflammatory disease	2	1.2		2	1.9		*
	**N**	**Median (range)**	**N**	**Median (range)**	**p**		
Time between symptom onset and diagnosis of JIA (months)	165	0.55 (0–8.78)	102	0.47 (0.1-10.26)			.19^c^
Disease duration (years)	165	3.46 (0.28-14.82)	102	3.77 (0.45-14.13)			.15^c^
Physician disease activity (VAS score range 0–100)	165	15.00 (0–84)	102	17.00 (0–94)			.85^c^
Number of joints with arthritis	**N**	**%**		**N**	**%**		**p**
No arthritis	76	45.2		46	43.8		.82^b^
Monoarthritis (1 joint)	21	12.5		18	17.1		.29^b^
Oligoarthritis (2–4 joints)	45	26.8		22	21.0		.28^b^
Polyarthritis (>4 joints)	23	13.7		19	18.1		.57^b^
Uveitis presenting during disease course	8	4.8		9	8.6		.45^b^
Medication at time point of evaluation	**N**	**%**		**N**	**%**		**p**
No medication	13	7.7		16	15.2		.05^b^
NSAID	98	58.3		47	44.8		.029^b^
DMARDS (including methotrexate, sulfasalazine)	131	78.0		76	72.4		.38^b^
Anti-TNF	24	14.3		1	9.5		*
DMARDS and Biological (Anti-TNF)	21	12.5		9	8.6		.31^b^

a = independent samples T-test, b = Chi^2^, c = Mann-Whitney U test, * = less than 5 cases in a cell.

Abbrevations: *JIA* = Juvenile Idiopathic Arthritis, *VAS* = Visual Analogue Scale, *NSAID* = Non Steroid Anti-Inflammatory Drug, *DMARD* = Disease Modifying Anti-Rheumatic Drug: methotrexate, sulfasalazine, *Anti-TNF* = anti-Tumor Necrosis Factor: etanercept, adalimumab, infliximab.

### Functional ability and discomfort

The CHAQ scores were available from 158 patients. The mean total score was 0.73 (SD 0.8). The mean patient and parent reported VAS pain score and general well-being was respectively 28.67 (SD 29.6) and 27.58 (SD 26.9).

Differences between children without active arthritis and with active arthritis were found on all three outcomes (Mann–Whitney U test); CHAQ mean total score (0.54, SD 0.6 vs 0.97, SD 0.7, p < .001), VAS pain score (17.6, SD 25.4 vs 38.2, SD 29.8, p < .001) and VAS score of general well-being (18.0, SD 23.7 vs 35.8, SD 26.9, p < .001).

### HRQOL

#### Parents of a child with JIA compared with the Dutch norm

Parents of a child with JIA differed from the Dutch norm on the following characteristics: parents of a child with JIA were on average younger (42.0 years, SD 5.4 vs 43.7 years, SD 5.5, p < .001), were more often married/living together (87.5% vs 83.1%, p < .001) and the distribution of parental educational level differed as well (p < .05). The HRQOL of parents of children with JIA, corrected for parental age and educational level, was comparable to the HRQOL of parents of healthy children on ten of the twelve subscales. Parents of children with JIA scored significantly worse (p < .05) on the subscale ‘fine motor functioning’ and significantly better (p < .05) on the subscale ‘social functioning’ (Table 2).

**Table 2:** HRQOL of parents of children with JIA compared with Dutch norm data

	**JIA parents (n = 167)**			**Norm group (n = 425)**			
**HRQOL**	**n**	**Mean**	**SD**	**n**	**Mean**	**SD**	**p**
Daily activities	167	83.53	24.95	420	85.26	21.37	.44^a^
Aggressive emotions	167	88.82	18.64	421	87.57	16.41	.17^a^
Cognitive functioning	167	79.90	26.30	421	78.74	24.69	.32^a^
Fine motoric functioning	167	92.37	19.63	422	96.82	9.14	<.001^a^
Gross motoric functioning	167	84.96	23.04	423	87.95	20.84	.30^a^
Pain	167	68.08	26.90	424	72.07	22.32	.16^a^
Positive emotions	167	67.96	21.99	422	66.57	19.93	.30^a^
Sexuality	167	87.28	23.14	405	84.72	23.58	.25^a^
Sleep	167	67.44	27.50	423	70.81	25.38	.19^a^
Social functioning	167	89.78	14.00	421	83.59	20.07	.001^a^
Depressive emotions	167	75.45	22.34	423	78.55	19.52	.18^a^
Vitality	167	60.53	24.62	423	63.36	22.63	.39^a^

a = Analysis of variance, with parental age and educational level as covariates.

#### Parents of a child with JIA without active arthritis compared with parents of a child with JIA with active arthritis

Parental educational level differed between parents of children with versus without active arthritis (p < .05). Corrected for their educational level, parents of children with active arthritis showed significantly (p < .01) worse HRQOL than parents of children without arthritis on the following subscales: daily activities, cognitive functioning and depressive emotions (Table 3).

**Table 3:** HRQOL of parents of children with JIA without active arthritis in comparison to parents of children with JIA with active arthritis

	**No arthritis (n = 76)**			**Arthritis (n = 92)**			
**HRQOL**	**n**	**Mean**	**SD**	**n**	**Mean**	**SD**	**p**
Daily activities	76	88.57	19.75	91	79.33	27.99	.03^a^
Aggressive emotions	76	89.33	15.55	91	88.40	20.95	.69^a^
Cognitive functioning	76	87.75	20.09	91	73.35	29.06	.001^a^
Fine motoric functioning	76	94.90	15.30	91	90.25	22.48	.19^a^
Gross motoric functioning	76	87.58	19.62	91	82.76	25.45	.33^a^
Pain	76	69.90	26.55	91	66.55	27.23	.65^a^
Positive emotions	76	70.29	22.95	91	66.03	21.09	.23^a^
Sexuality	76	87.01	23.93	91	87.50	22.59	.91^a^
Sleep	76	71.71	26.23	91	63.87	28.16	.08^a^
Social functioning	76	91.28	13.70	91	88.53	14.20	.27^a^
Depressive emotions	76	80.26	20.22	91	71.43	23.31	.02^a^
Vitality	76	64.58	22.78	91	57.14	25.69	.11^a^

a= Analysis of variance, with educational level as covariate.

### Parental perceptions of child vulnerability

#### Parents of a child with JIA compared with the Dutch norm

Parents of a child with JIA perceived their child as more vulnerable compared with parents of a healthy child. Significant differences (p < .001) were found on the total CVS score, on all items of the CVS, and on the percentages of parents with scores above the cut-off (scores ≥ 10); 31% of parents of a child with JIA vs 1% of parents of a healthy child.

Compared with parents of a chronically ill child, parents of a child with JIA also perceived their child as significantly (p < .001) more vulnerable as expressed both the total score, and by the percentage above the cut-off score; 31% vs 7.2% (p < .001). Significant differences were also found on five of the eight items (Table 4).

**Table 4:** Results on the Child Vulnerability Scale (CVS) in parents of children with JIA, compared with a reference group of Dutch parents of both healthy and chronically ill children

	**JIA parents (N = 168)**		**Reference group healthy child (N = 450)**			**Reference group chronically ill child (N = 69)**		
**CVS**	**Median (25-75%)**		**Median (25-75%)**		** p**	**Median (25-75%)**		** p**
Total score CVS - range 0-24	6 (3–11)		1 (0–3)		<.001^b^	4 (1–6)		<.001^b^
	**N**	**%**	**N**	**%**	**p**	**N**	**%**	**p**
Above cut-off score	52	31.0	5	1.1	<.001^a^	5	7.2	<.001^a^
Items, *(mostly) true*:	**N**	**%**	**N**	**%**	**p**	**N**	**%**	**p**
1. In general, my child seems less healthy than other children.	52	31.0	9	2.0	<.001^a^	6	8.7	<.001^a^
2. I often think about calling the doctor about my child.	22	13.1	4	0.9	<.001^a^	6	8.7	.340^a^
3. When there is something going around my child usually catches it.	50	29.8	26	5.8	<.001^a^	9	13.0	.008^a^
4. I often check on my child at night to make sure that s/he is okay.	40	23.8	37	8.2	<.001^a^	13	18.8	.404^a^
5. Sometimes I get concerned that my child doesn’t look as healthy as s/he should.	55	32.7	15	3.3	<.001^a^	9	13.0	.002^a^
6. I often have to keep my child indoors because of health reasons.	16	9.5	1	0.2	<.001^a^	6	8.7	.842^a^
7. I get concerned about circles under my child’s eyes.	40	23.8	13	2.9	<.001^a^	8	11.6	.034^a^
8. My child gets more colds than other children I know.	50	29.8	17	3.8	<.001^a^	10	14.5	.014^a^

a = Chi^2^, b = Mann-Whitney U test.

Higher scores represent more perceived vulnerability of the child.

NOTE: Cut-off point high perceived vulnerability: total score of 10 and above [28].

#### Parents of a child with JIA without active arthritis compared with parents of a child with JIA with active arthritis

Significant differences (p < .05) were found on the total CVS score between parents of children with active arthritis (median 7, range 4–12) and parents of children without active arthritis (median 5, range 2–9). No differences were found on the percentages of parents with scores above the cut-off (scores ≥ 10) or on the items of the CVS, except for item 1; “In general, my child seems less healthy than other children” (96.2% for parents of children with active arthritis vs 30.8% for parents of children without active arthritis, p < .05).

#### Identifying variables associated with parental perceptions of child vulnerability

The results of the final logistic regression analysis are shown in Table 5. The CHAQ total score (functional ability) and disease duration were significantly associated with PPCV (p < .05). Parents of a child with higher scores on the CHAQ total score, perceived their child as more vulnerable. Shorter disease duration is associated with a more vulnerable perception of the child. Parental age, number of active joints and pain were not significantly related to PPCV.

**Table 5:** Logistic regression model of parental perceptions of child vulnerability predicted by parental and child variables

**N = 145**	**OR**	**95% confidence interval**	**p**
Parental age	.96	[.88-1.04]	.29
Number of active joints	1.39	[.96-2.01]	.08
Disease duration	.81	[.68-.98]	.03
Functional disability (CHAQ total score)	3.23	[1.39-7.53]	<.01
Pain (VAS scores)	.99	[.98-1.02]	.95

## Discussion

This study shows that the HRQOL of parents of a child with JIA was comparable to the HRQOL of parents of healthy children. The only differences we found were that parents of a child with JIA scored worse on ‘fine motor functioning’ and better on ‘social functioning’.

These results are in line with some previous research regarding HRQOL of parents of a child with JIA [26]. The normal levels of HRQOL might be explained by the multidisciplinary therapeutic approach in children with JIA and good education of the parents regarding the course and outcome of the illness [26]. In the Netherlands, treatment is coordinated by regular checks by a pediatric rheumatologist and ophthalmologist. Occupational and physical therapy are integral in the management of JIA to improve mobility and help to manage pain. Psychosocial support for the patient and family by a psychologist and social worker is often organized to improve self-management and coping with the impact of the illness in daily life.

The lowered fine motor functioning could be related to the findings that JIA occurs more often in families with presence of other autoimmune or rheumatic diseases [38,39]. It was not possible to control for this aspect, because data about the presence of a parental chronic illness were not available.

Earlier, we studied the HRQOL of the children with JIA from the parents in the present study [40]. The children reported a worse HRQOL compared with their peers. It is remarkable that the HRQOL of parents is not affected while the children reported very poor HRQOL, because it is known that children’s well-being and parents’ HRQOL and psychosocial functioning are associated. When children are chronically ill, parents are often more distressed and more at risk for a lower HRQOL compared to parents of healthy children [7,41]. Moreover, high levels of distress in parents are associated with the child’s maladjustment to the illness [15-18].

It is questionable whether a generic HRQOL measurement such as the TAAQOL which was used in our study, is sensitive enough to measure HRQOL in parents of a chronically ill child or that specific measurements for parents of a chronically ill child would detect HRQOL problems better. Future research should focus on the relationship between the HRQOL of children with JIA and the HRQOL of their parents.

On the other hand, parents of a child with JIA with active arthritis showed a worse HRQOL compared with parents of a child with JIA without arthritis; especially on the subscales ‘daily activities’, ‘cognitive functioning’, and ‘depressive emotions’. Although results of previous studies are unclear about the relationship between a child’s disease severity and parental HRQOL, our findings suggest the influence of an active disease status on the parental HRQOL.

Parents of a child with JIA perceived their child as more vulnerable compared with parents of a healthy child and compared with parents of a child with other chronic health conditions. This is in line with findings in other patient groups [31,42] and in a previous study on parents of a child with JIA [29]. An important question is whether the scores are excessively high or a good reflection of the child's vulnerability, because children with JIA are "truly vulnerable" compared with healthy children. The perceptions of the parents to perceive their children with JIA as more vulnerable than healthy children may be realistic [28], because children with JIA do have a chronic illness and are in some way more vulnerable in having doctors’ visits, looking less healthy and staying at home because of health complaints. However, it is known that parents who perceive their chronically ill child as more vulnerable are more likely to overprotect their child [43]. This overprotection may hamper the child to develop the personal skills needed to cope with the challenges of growing up with a chronic disease. Therefore, health care providers should encourage parents to stimulate the independence of their child [44].

Parents of a child with JIA with active arthritis perceived their child overall as more vulnerable compared with parents of a child with JIA without arthritis, as indicated by the total score for perceived vulnerability. In addition, they think more often that their child seems less healthy than other children. These findings suggest an influence of disease activity on parental perceptions of child vulnerability.

To get more insight in the PPCV, we aimed to identify variables associated with the PPCV. Our study shows that parents of a more functionally disabled child report to perceive their child as more vulnerable. When the disease duration is shorter, parents also perceive their child as more vulnerable. It is known that for parents, the first confrontation with the knowledge that their child has a chronic illness is a stressful and potentially traumatic event. After the initial shock of the diagnosis, the impact of the disease and its treatments on the child and family may gradually become more clear and results in less worrying thoughts in parents [45].

The findings of this study are qualified by several limitations, including the analysis performed to identify parental and child variables associated with PPCV. Because of multicollinearity, the VAS general well-being and physician disease activity could not be included in the final logistic regression model of PPCV, while these factors appeared to be correlated with PPCV in the univariate logistic analyses.

Regarding the differences we found on parental perceptions of child vulnerability between JIA parents and parents of children with a chronic illness in the norm population, the following should be taken into account. It is plausible that the more severely ill children were not included in the norm population, since the sample for the norm population was collected from regular schools. Because the identification of children with a chronic health condition was based on parental report, objective information on disease severity was not available.

This study analyzed cross-sectional data, which makes it impossible to draw any conclusions about the causality between the associated parental and child variables with PPCV.

The medical data of the non-participating children were collected retrospectively and the parental country of birth was retrieved from the medical records. Based on this data the participants differed significantly from the non-participants on parental country of birth, child’s age and medication. Therefore, it is hard to generalize these results to the whole group parents with a child with JIA. The literature on the psychosocial impact of parents of children with JIA is inconsistent; some studies reported significant psychosocial impact on parents, while others found no differences in parents of a child with JIA compared with normative data. Most studies are retrospective reports, consist of a relatively small sample of children or parents [46] and were published more than a decade ago. Therefore, these data may not be representative for the current group of children with JIA and their parents, since the treatment of children with JIA has significantly improved over recent years [1]. Due to these treatment changes, it is to be expected that more children and adolescents with JIA will have a better HRQOL and less overall burden of the disease over time. As a consequence, parents may also experience less psychosocial problems.

Our study contributes to the existing literature on functioning of parents of a child with JIA because of the relatively large study sample and the inclusion of two important outcomes in the parental functioning and care for a chronically ill child; HRQOL and PPCV. A strong point of this study is that we did not only focus on outcomes of parental functioning, but also on the determinants. We used a website for data collection. Using the Internet for participants to complete the questionnaires is valid and reliable and less time-consuming compared to a paper and pencil version, and with less missing data.

## Conclusions

In conclusion, there is a need for further research on the HRQOL, psychosocial functioning, needs and PPCV in parents of a child with JIA to gain knowledge on the starting points for developing interventions for these parents to improve their well-being, and also their child’s well-being. A recent review about psychological interventions for parents of children with a chronic illness shows that psychological interventions for parents can help reduce pain in children with painful conditions [46]. It is well-known that pain in children with JIA predicts their HRQOL [40]. Therefore, children’s HRQOL could be improved through psychological interventions for their parents. So, while caring for their child is the first priority, parent’s potential burden should be recognized, as well as their stress levels and reactions to the uncontrollable aspects of the illness. To improve the care for children with JIA, more attention should be paid to research on the parents.

### Key messages

• The HRQOL of parents of a child with JIA was comparable to the HRQOL of parents of a healthy child.

• Parents of a child with active arthritis showed worse HRQOL than parents of a child without active arthritis.

• Parents perceived their child with JIA as more vulnerable than parents of a healthy child or parents of a child with other chronic health conditions.

• Parents perceived their child as more vulnerable when their child with JIA was more functionally disabled and had a shorter disease duration.

## Abbreviations

ANOVA: Analysis of variance; CVS: Child Vulnerability Scale; CHAQ: Childhood Health Assessment Questionnaire; HRQOL: Health Related Quality of Life; ILAR: International League of Associations for Rheumatology; JIA: Juvenile Idiopathic Arthritis; PPCV: Parental perceptions of child vulnerability; SPSS: Statistical Package for the Social Sciences; TAAQOL: TNO-AZL Questionnaire for Adult’s Health Related Quality of Life; VAS: Visual Analogue Scale.

## Competing interests

The authors declare that they have no competing interests.

Pfizer BV Pharmaceuticals and AGIS Healthcare had no role in the study design, data collection, data analysis, or writing of this manuscript. Publication of this article was not contingent on the approval of these study sponsors.

## Authors’ contributions

LH made substantial contributions to the conception and design of the study and to the interpretation of data, collected the data, performed the analyses, helped to draft the article or to review it critically for important intellectual content, and gave final approval of the version to be published. HvO made substantial contributions to the interpretation of data, performed the analyses, helped to draft the article or to review it critically for important intellectual content, and gave final approval of the version to be published. HMS made substantial contributions to the conception and design of the study and to the interpretation of data, performed the analyses, helped to draft the article or to review it critically for important intellectual content, and gave final approval of the version to be published. TW made substantial contributions to the conception and design of the study and to the interpretation of data, helped to draft the article or to review it critically for important intellectual content, and gave final approval of the version to be published. MG made substantial contributions to the conception and design of the study and to the interpretation of data, helped to draft the article or to review it critically for important intellectual content, and gave final approval of the version to be published. MvR made substantial contributions to the conception and design of the study and to the interpretation of data, helped to draft the article or to review it critically for important intellectual content, and gave final approval of the version to be published. All authors read and approved the final manuscript.
